# Visualization of Flow‐Induced Strain Using Structural Color in Channel‐Free Polydimethylsiloxane Devices

**DOI:** 10.1002/advs.202204310

**Published:** 2022-11-17

**Authors:** Kota Shiba, Chao Zhuang, Kosuke Minami, Gaku Imamura, Ryo Tamura, Sadaki Samitsu, Takumi Idei, Genki Yoshikawa, Luyi Sun, David A. Weitz

**Affiliations:** ^1^ Center for Functional Sensor & Actuator (CFSN) Research Center for Functional Materials National Institute for Materials Science (NIMS) 1‐1 Namiki Tsukuba Ibaraki 305‐0044 Japan; ^2^ Department of Physics and John A. Paulson School of Engineering and Applied Sciences Harvard University 9 Oxford Street Cambridge MA 02138 USA; ^3^ Materials Science and Engineering Graduate School of Pure and Applied Science University of Tsukuba 1‐1‐1 Tennodai Tsukuba Ibaraki 305‐8571 Japan; ^4^ International Center for Materials Nanoarchitectonics (MANA) National Institute for Materials Science (NIMS) 1‐1 Namiki Tsukuba Ibaraki 305‐0044 Japan; ^5^ Graduate School of Information Science and Technology Osaka University 1‐2 Yamadaoka Suita 565‐0871 Japan; ^6^ Graduate School of Frontier Sciences The University of Tokyo 5‐1‐5 Kashiwanoha Kashiwa Chiba 277‐8568 Japan; ^7^ Research and Services Division of Materials Data and Integrated System (MaDIS) National Institute for Materials Science (NIMS) 1‐2‐1 Sengen Tsukuba Ibaraki 305‐0047 Japan; ^8^ Department of Applied Chemistry Faculty of Science and Technology Chuo University 1‐13‐27 Kasuga, Bunkyo‐ku Tokyo 112‐8551 Japan; ^9^ Polymer Program Institute of Materials Science and Department of Chemical & Biomolecular Engineering University of Connecticut Storrs CT 06269 USA; ^10^ Wyss Institute for Biologically Inspired Engineering Harvard University 3 Blackfan Street Boston MA 02115 USA

**Keywords:** gas sensor, plasma, polydimethylsiloxane, strain, structural color, wrinkles

## Abstract

Measuring flow of gases is of fundamental importance yet is typically done with complex equipment. There is, therefore, a longstanding need for a simple and inexpensive means of flow measurement. Here, gas flow is measured using an extremely simple device that consists of an Ar plasma‐treated polydimethylsiloxane (PDMS) slab adhered on a glass substrate with a tight seal. This device does not even have a channel, instead, gas can flow between the PDMS and the glass by deforming the PDMS wall, in other words, by making an interstice as a temporary path for the flow. The formation of the temporary path results in a compressive bending stress at the inner wall of the path, which leads to the formation of well‐ordered wrinkles, and hence, the emergence of structural color that changes the optical transmittance of the device. Although it is very simple, this setup works sufficiently well to measure arbitrary gases and analyzes their flow rates, densities, and viscosities based on the change in color. It is also demonstrated that this technique is applicable to the flow‐induced display of a pattern such as a logo for advanced applications.

## Graphical abstract

It is demonstrated that a super simple device consisting of a glass and a plasma‐treated polydimethylsiloxane (PDMS) is useful for measuring gases based on the change in color caused by flow‐induced deformation of the PDMS. This technique allows to analyze flow rate, density, and viscosity of the gases, as well as, to display an arbitrary pattern such as a logo.

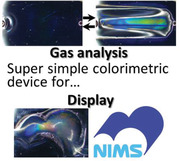

## 1 Introduction

Measuring flow of gases is a central challenge in every fluid‐related field. Many methods exist for measuring not only air flow but also flows of various gases, typically using dedicated, well‐developed equipment.^[^ ^1^ ^]^ By contrast, far fewer methods exist that enable simple measurements of flow using compact and inexpensive equipment. Such devices would significantly broaden the applications and utility of flow measurements for fluids. Among various approaches, measuring fluid flow is frequently reduced to determining the pressure required to achieve a given flow rate. This is accomplished by using a pressure gauge which is often cumbersome both in the setup and in the readout. Typical pressure gauges function by measuring the strain induced by the pressure; thus, the problem is reduced to measuring a strain. In addition to a wide variety of commercial strain gauges, several tactile sensors are available to measure strain.^[^ ^2^ ^]^ In addition, many advanced applications demand a 2D measurement of the pressure, and hence of the strain. For this purpose, digital image correlation and related techniques can be used;^[^ ^3^ ^]^ they can visualize how strain is distributed over an entire flow channel. Although these techniques are powerful and well‐developed, they rely on a bulky and expensive setup for the measurements. For example, a polydimethylsiloxane (PDMS)‐based microchannel combined with stimuli‐responsive coloration has been developed for pressure sensing. Various means of detecting the change in color have been proposed including photonic crystal gratings,^[^ ^4^ ^]^ lenses,^[^ ^5^ ^]^ interference,^[^ ^6^ ^]^ dyed solutions,^[^ ^7^ ^]^ and pressure‐sensitive pigments.^[^ ^8^ ^]^ However, fabricating such functional microchannels requires multiple steps with special facilities in a clean environment,^[^ ^9^ ^]^ while the measurement of the strain typically requires much more sophisticated apparatus. Therefore, the development of a greatly simplified and facile technique would contribute to the realization of a mobile flow sensor that could monitor the flow and discriminate various gases by quantifying their properties.

In this paper, we describe an approach to measuring flow of gases through the visualization of the flow‐induced strain using a simple PDMS‐based device that does not even have a channel for the flow. We accomplish this by treating a PDMS slab with two plasma: oxygen (O_2_) and argon (Ar). An O_2_ plasma is typically used to induce covalent bonds between PDMS and a substrate including glass when adhering a cover to a device; by contrast, an Ar plasma barely induces any covalent bonding, but instead modifies the PDMS surface to form a more rigid glass‐like layer on its surface. The contrast in the elasticity between this rigid topmost layer and the softer bottom material causes a well‐ordered wrinkle structure to form when the PDMS is deformed. Since the wavelength of the induced wrinkles is in the range of a few microns, we observe an angle‐dependent structural color upon deformation of the PDMS. To fabricate a device to measure flow, we use a mask to locally treat a PDMS slab with Ar plasma and then use a negative mask to treat the rest of the slab with an O_2_ plasma. Thus, the PDMS forms covalent bonding with a glass substrate except where it has been treated by the Ar plasma, where it remains chemically unattached; this allows gases to flow only between this area and the substrate. Such a geometry, without pre‐formed channels, maximizes the deformation of the PDMS due to the flow of a gas. This deformation can be measured by visualizing the structural color of the wrinkle pattern induced by the deformation, providing a very simple and compact means of measuring the pressure of the flowing gas. With this PDMS device, we show that flow rate of gases can be measured by quantifying the change in color. In addition, we discuss the dependence of the color on density and viscosity of the gases. We also demonstrate that the technique is capable of displaying a specific pattern such as a logo under gas flow.

## 2 Results and Discussion

The basic principle used in the creation of our devices is the treatment of a PDMS with a gas plasma. The surface of PDMS microfluidic devices is commonly exposed to an O_2_ plasma which makes the interface reactive and improves the bonding to a glass substrate; this method is commonly employed to improve the seal of a microfluidic device.^[^ ^9^ ^]^ Another consequence of treatment with an O_2_ plasma is to create a thin, stiff layer on the surface of the PDMS.^[^ ^10^ ^]^ The stiffness of this topmost layer is much greater than that of the PDMS.^[^ ^11^ ^]^ Treatment of a PDMS surface with an Ar plasma also leads to the creation of a very stiff, thin layer at the surface,^[^ ^12^ ^]^ but without the concomitant formation of an adhesive surface. Therefore, surfaces treated with an Ar plasma can be easily peeled from the glass substrate and do not stick to the substrate in the same manner as those treated with an O_2_ plasma. We use this feature to pattern a PDMS surface with adhesive regions which will bond to a glass substrate and non‐adhesive regions which do not bond to the glass yet are much stiffer than the bulk PDMS.

A thin, stiff layer of PDMS atop a much less stiff region also has another valuable feature that is observed upon compressing the PDMS. Since the thin, stiff layer is much more resistant to compression than the bulk PDMS, it undergoes a buckling transition which induces nearly periodic wrinkles in the surface.^[^ ^13^ ^]^ These wrinkles have a spacing of around a micron, which is comparable to the wavelength of light; this creates a diffractive grating structure, causing an easily detectable diffraction of light at specific wavelengths. Moreover, both the spacing and the amplitude of these wrinkles are very sensitive to the compression of the PDMS, and hence to the strain applied to the PDMS in other words. This provides a simple yet highly sensitive means of measuring the strain on the PDMS. If this strain is caused by the flow of gas, it also becomes a sensitive means to detect this flow.

**Figure 1:**
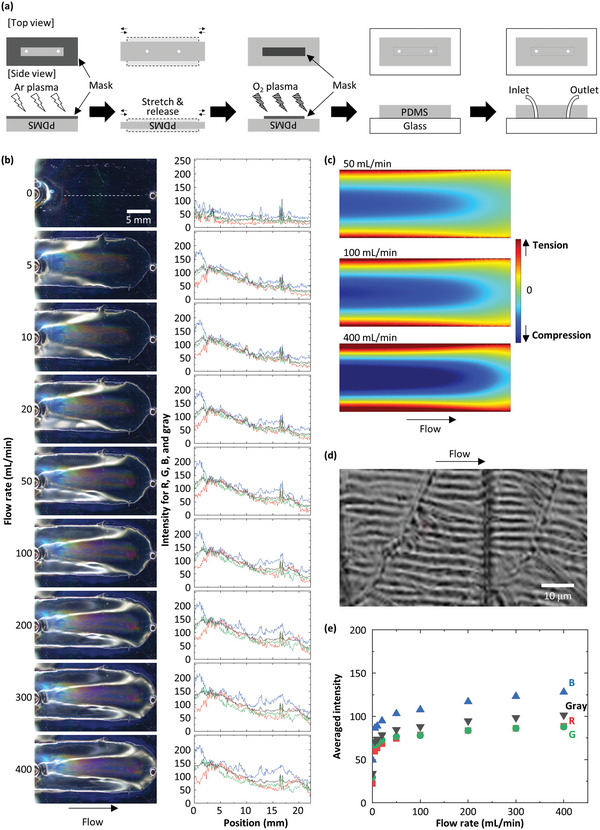
Channel‐free PDMS device fabrication and gas measurements. a) Schematic of the device fabrication. Both top and side views are shown at each step. b) Photos of the device taken under the flow of N_2_ at 0, 5, 10, 20, 50, 100, 200, 300, and 400 mL min^−1^. Intensity profiles for red (R), green (G), blue (B), and gray are also shown right next to the corresponding photos. These profiles are obtained by slicing the photos along the white dashed line only shown at 0 mL min^−1^. c) Results of FEA simulations that show the stress in *y‐*direction (across the flow) exerted on the inner wall of the PDMS slab induced by N_2_ flows at 50, 100, and 400 mL min^−1^ (top to bottom). Note that the simulated geometry is identical to that in (b) except an open space (50 µm in height) between the top PDMS and the bottom glass is assumed here to facilitate the simulation. d) Laser microscope image of the wrinkles formed under the flow of N_2_ at 10 mL min^−1^. This image is recorded at the middle of the flow path. e) Plot of averaged intensities for R, G, B, and gray as a function of flow rate. These values are obtained by averaging the intensities shown in (b).

To realize such compression‐induced wrinkling and the resultant change in color under flow, we locally treat a PDMS slab with both O_2_ and Ar plasma using both positive and negative masks to prepare regions with different adhesivity in the PDMS, as shown in **Figure** **1**a. Since the Ar plasma‐treated part can be peeled off even after the entire PDMS is adhered to a glass substrate, gas injection will deform the Ar plasma‐treated PDMS and create a temporary interstice between the Ar plasma‐treated area and the glass. Importantly, such a geometry, without pre‐formed channels, maximizes the deformation of the PDMS due to the flow of the gas. To implement this idea, the central portion of the PDMS slab (≈2.5 mm in thickness) is exposed to an Ar plasma through a positive mask, as shown in dark gray in Figure 1a. The PDMS slab is stretched with ≈20% strain for a few seconds after the Ar plasma treatment to intentionally induce cracks in a controlled fashion. This process is necessary for reproducible fabrication because it prevents uncontrolled crack formation caused by unexpected bending or stretching during the fabrication process. Similar to O_2_ plasma‐treated PDMS,^[^ ^14^ ^]^ the Ar plasma‐treated PDMS exhibits a brilliant angle‐dependent color when it is stretched. Then, we use a negative mask to cover the Ar plasma‐treated area and expose the remaining surface to an O_2_ plasma, as shown in Figure 1a. The PDMS slab is then adhered to a glass substrate to complete the device fabrication.

To test the functioning of the device, we inject nitrogen (N_2_) gas at a flow rate of 400 mL min^−1^ into the device; we observe structural color in the Ar plasma‐treated area as expected, as shown in Figure 1b. Simulations using finite element analysis (FEA) reveal that this area overlaps with the area that undergoes compression due to the pressure load of the gas flow, which suggests that it is the compression that drives the formation of wrinkles, and hence, the emergence of structural color, as shown in Figure 1c. This explanation is also supported by the fact that the profile of the structural color varies symmetrically along the direction perpendicular to the flow, which is in excellent agreement with the compression profile obtained by the FEA. The presence of the wrinkles is confirmed by observing the device under the flow with a microscope. The wrinkles are oriented along the flow direction, and the wavelength is estimated to be roughly 2–3 µm, as shown in Figure 1d. Some oblique cracks are also observed, which should be formed as a result of the deformation that occurs along two directions: the flow direction and the direction perpendicular to the flow. Although this crack formation is inevitable, the number of the cracks does not significantly change once they form. Therefore, measurements and analyses can be done in a reproducible fashion even in the presence of the oblique cracks. To investigate how the gas flow correlates with the structural color, we examine the change in color by changing flow rate over the range of 5–400 mL min^−1^. The color is already observable at flow rates even as low as 5 mL min^−1^; it then becomes brighter as the flow rate increases. We extract red (R), green (G), blue (B), and gray (average of RGB) values from each image and plot them for more quantitative analyses. Clearly, the averaged intensities for R, G, and B increase as the flow rate increases, as shown in Figure 1e. This result indicates that a higher flow rate will induce a greater deformation of the PDMS, which leads to a larger amplitude of the wrinkles and a larger change in color. This observation is confirmed by the FEA simulations, as shown in Figure 1c.

**Figure 2:**
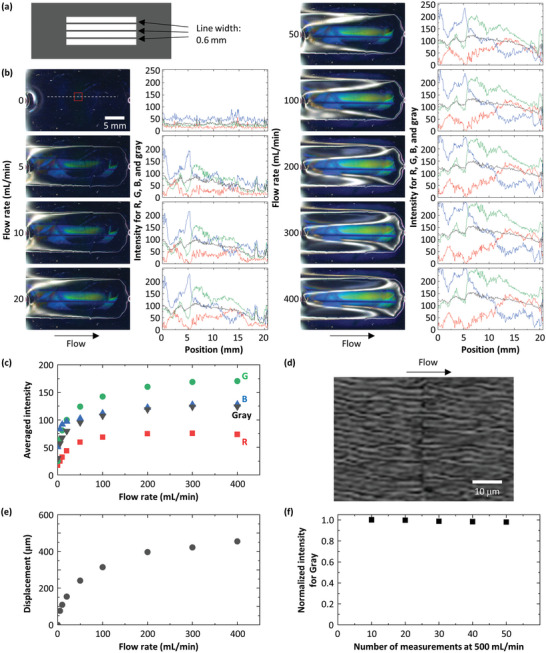
Modified PDMS device fabrication and gas measurements. a) Schematic of a mask used to fabricate the modified device. b) Photos of the modified device taken under the flow of N_2_ at 0, 5, 10, 20, 50, 100, 200, 300, and 400 mL min^−1^. Intensity profiles for R, G, B, and gray are also shown right next to the corresponding photos. These profiles are obtained by slicing the photos along the white dashed line only shown at 0 mL min^−1^. c) Plot of averaged intensities for R, G, B, and gray as a function of flow rate. These values are obtained by averaging the intensities along the dashed line shown in (b). d) Laser microscope image of the wrinkles formed under the flow of N_2_ at 10 mL min^−1^. This image is taken at the middle of the flow path shown in (b) with a red square. e) Plot of PDMS displacement as a function of flow rate. The displacement is recorded at the middle of the flow path shown in (b) with a red square. f) Plot of normalized intensities for gray measured at 500 mL min^−1^ as a function of the number of measurements.

Introducing defects can enhance the regularity of wrinkles and the sensitivity of the device. For this purpose, the positive mask used in the device fabrication is replaced with a modified mask. This mask has three thin lines evenly spaced along the flow direction with a width of ≈0.6 mm, as shown in **Figure** **2**a. The areas beneath the lines are expected to be protected from the Ar‐plasma exposure, which is confirmed by observing the border between plasma‐treated and non‐treated areas, as shown in Figure S1, Supporting Information. The protected areas can work as defects that will lead to the formation of more regularly spaced wrinkles,^[^ ^15^ ^]^ and hence, a more pronounced structural color. We confirm that the structural color in this device shows higher intensities than that in the first device without the lines, as shown in Figure 2b,c. Moreover, this device produces denser wrinkles with higher regularity, as observed by comparing Figure 2d to Figure 1d. Regardless of the enhanced regularity and color, the new device shows the same trend as the first one where the color becomes brighter as the flow rate increases and measures the structural color over a wide range of flow rate from several mL min^−1^ to several hundred mL min^−1^. The flow‐rate dependence of the change in color intensity correlates with the PDMS displacement, as shown in Figure 2e. Here, the displacement is estimated by first taking an image without flow and then measuring how much the height must be adjusted to obtain a focused image under different flow rates. Although the displacement increases monotonically but non‐linearly as a function of flow rate, sensitivity of this measurement is roughly calculated to be 1 µm per unit flow rate by assuming linearity and using the displacement measured at 400 mL min^−1^. This is a universal form of sensitivity that describes the present device; in addition to the optical detection based on structural color using a CCD, other analytical techniques can be also used to evaluate and improve the performance. In a low magnification, a vivid color and its gradation are observed by changing lighting conditions, as shown in Figure S2, Supporting Information. By contrast, the device without the lines shows a rather sparse color profile, as shown in Figure 1b. These results confirm that the introduction of the lines helps the wrinkles form more densely in a limited area, leading to the enhanced color that enables sensitive measurements.

In addition to sensitivity, we also test other important characteristics of the device including stability and measurement error. We use the same device, and N_2_ flow is on and off every 10 s at least 50 times. Data are collected every 10 cycles. As a result, there is no significant change in normalized intensities after the multiple cycles, as shown in Figure 2f. In these measurements, flow rate is set at 500 mL min^−1^, which is even higher than that we use in the experiments described above. The measurement error is estimated to be as low as 1%, indicating sufficient robustness and reliability of the device for repeated use.

**Figure 3:**
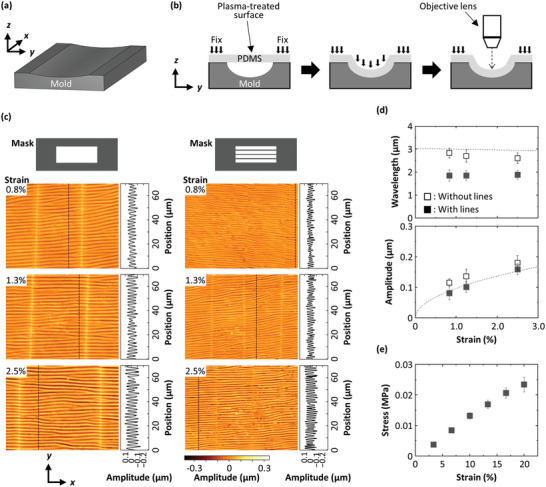
Characterization of wrinkles formed under compression on a curved surface. a) Illustration of a 3D‐printed mold. b) Schematic to show how a PDMS slab is adhered to the mold. c) Images of the PDMS taken under strains of 0.8%, 1.3%, and 2.5% using the molds with different curvatures. The amplitude profiles are recorded along the dashed line in each image. The left three and right three data are obtained from two PDMS slabs that are plasma‐treated with different masks: (left) without and (right) with lines. d) Plots of (top) wavelength and (bottom) amplitude of the wrinkles as a function of strain. These values are obtained from the amplitude profiles shown in (c). The dotted lines are the analytic models described in the main text as Equations (1) and (2). e) Stress–strain relationship of the PDMS. All error bars represent standard deviation.

To describe the wrinkle formation in our device in more detail, we fabricate a mold with a curved surface, as shown in **Figure** **3**a,b. The curved shape of the PDMS mold is designed to mimic the top wall of a microchannel deformed by the pressure that drives flow through the channel.^[^ ^16^ ^]^ This curved cross‐section should be useful in reproducing the wrinkles formed in our device under gas flow, and hence, in estimating how much bending strain is generated. For this purpose, we perform FEA simulations, assuming that the flow‐induced deformation is approximately reproduced by applying a uniform load on one side of a PDMS slab with both its ends fixed, as shown in Figure S3, Supporting Information. As a result, the bending strains of the PDMS are estimated to be approximately 1% to 4%. Thus, we fabricate three molds with different curvatures that induce equivalent bending strains of 0.8%, 1.3%, and 2.5%. By putting the Ar‐plasma treated PDMS on these curved molds, we observe the formation of wrinkles at a bending strain as low as 0.8%, and they have a wavelength that is identical to that observed under flow of N_2_ at 10 mL min^−1^ (≈0.8% strain), as shown in Figure 3c and Figure S4, Supporting Information. The wavelength is almost constant at around 2.8 µm and decreases slightly as the strain increases, whereas the amplitude monotonically increases as a function of bending strain, as shown in Figure 3d. The wrinkles are consistent in multiple locations, as shown in Figures S5 and S6, Supporting Information. The wavelength *λ* and the amplitude *A* of the wrinkles formed on a curved surface are calculated by

(1) $$\frac{\lambda }{\lambda_{0}}=1-\varepsilon$$

(2) $$A=\frac{\lambda_{0}}{\pi }\;\sqrt{\varepsilon }$$

with *λ*_0_ is defined as

(3) $$\lambda_{0}=2\pi {\left[\frac{E_{f}t_{f}^{3}\left(1+\nu \right)\left(3-4\nu \right)}{E_{s}\left(1-\nu \right)}\right]}^{\frac{1}{3}}$$

where ε is the strain, *E*_f_ and *E*_s_ are the Young's moduli of the topmost thin layer of PDMS which is formed by an Ar plasma and the bulk PDMS underneath the thin layer, respectively, *ν* is the Poisson's ratio of PDMS, and *t*_f_ is the thickness of the top thin film.^[^ ^17^ ^]^ Here we assume that *E*_f_ = 0.4 GPa and *t*_f_ = 23 nm.^[^ ^18^ ^]^ These values are determined for PDMS treated with an O_2_ plasma with similar values of the plasma power, pressure, and duration. We use *ν* = 0.499, which is a typical value.^[^ ^19^ ^]^ We experimentally determine *E*_s_ to be 0.13 MPa, as shown in Figure 3e. The analytic solutions agree well with the experimental results obtained with the plasma‐treated PDMS using the mask without the lines. In general, the amplitude increases as the strain increases, while the wavelength of the wrinkles is almost constant at around 1.9 µm regardless of strain, as shown in Figure 3d. This difference in wavelength, 2.8 and 1.9 µm, is exactly what we observe under flow using the two devices.

**Figure 4:**
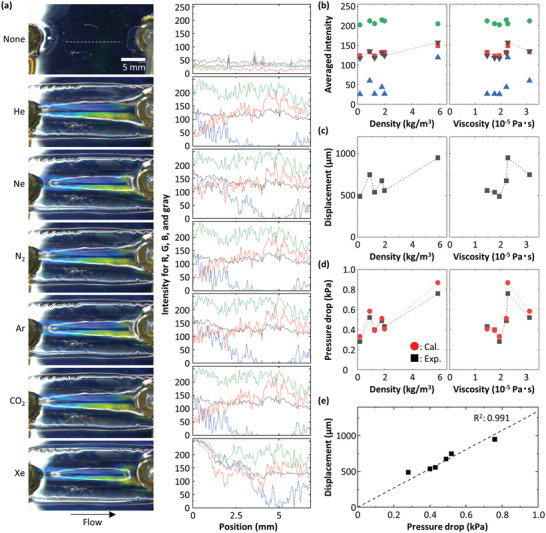
Measurements of various gases using the modified device. a) Photos of the device taken under the flow of various gases including He, Ne, N_2_, Ar, CO_2_, and Xe at 400 mL min^−1^. Intensity profiles for R, G, B, and gray are also shown right next to the corresponding photos. These profiles are obtained by slicing the photos along the white dashed line only shown at 0 mL min^−1^. b) Plot of averaged intensities for R, G, B, and gray as a function of densities (left) and viscosities (right). c) Plot of displacements as a function of densities (left) and viscosities (right). d) Plot of pressure drops as a function of densities (left) and viscosities (right). The black squares and the red circles show experimental data and calculated values, respectively. e) Plot of displacements as a function of pressure drops. The dashed line is the result of linear fitting.

The change in color is also affected by other factors, such as density and viscosity of gas. To evaluate their impacts on the measurement results, we measure the color changes in the device fabricated with the three parallel lines using six inert gases: Helium (He), neon (Ne), N_2_, Ar, carbon dioxide (CO_2_), and xenon (Xe) at a fixed flow rate of 400 mL min^−1^ at 20 °C. The densities and the viscosities of the six gases are listed in **Table** **1**. The difference in color intensity and pattern is not very pronounced among the six gases; nevertheless, there is a recognizable difference, particularly between He and Xe, as shown in **Figure** **4**a. To quantify these results, we calculate averaged RGB intensities for each of them. The data exhibit a small, but nevertheless discernable dependence on both viscosity and density, as shown in Figure 4b. To better quantify the response of the device, we measure the displacement of the PDMS at the middle of the flow path for each of the six gases. The dependence of the displacement is similar to that of the intensity, albeit exhibiting a more pronounced variation with both density and viscosity of the gases, as shown in Figure 4c. Interestingly, the pressure drop, Δ*p*, which is the difference between the pressure measured at the inlet and the outlet, also exhibits the same trend, including the discrete variations, as shown in Figure 4d. Importantly, the color intensities averaged over RGB exhibit a nearly identical trend as both the density and the viscosity, including the detailed gas‐to‐gas variations, but with a significantly reduced total variation. The detailed comparison is shown in Figure S7, Supporting Information. The reduced variation is presumably due to the strongly non‐linear response of the device; the variations of both the intensity and the displacement level off at higher flow rates, as highlighted in Figure 2c,e. All these measurements are made at higher flow rates, well within the range where they saturate, and the variations are reduced.

**Table 1:** List of densities and viscosities for the six gases.^[^ ^20^ ^]^

	Density [0 °C, 1 atm], *ρ* [kg m^−3^]	Viscosity [20 °C], *µ* [10^−5^ Pa s]
He	0.1785	1.95
Ne	0.9002	3.13
N_2_	1.251	1.76
Ar	1.783	2.22
CO_2_	1.977	1.47
Xe	5.851	2.28

To account for the dependence of pressure drop on gas properties, we use the modified Bernoulli's equation for steady, non‐uniform, incompressible viscous flow:^[^ ^21^ ^]^

(4) $$p_{1}+\frac{1}{2}\rho v_{1}^{2}+\rho g\;z_{1}=p_{2}+\frac{1}{2}\rho v_{2}^{2}+\rho gz_{2}+w_{\mu }$$

where *p* is the pressure, *ρ* is the density, *v* is the velocity, *g* is the acceleration of gravity, and *z* is the elevation. *w*_*μ*_ denotes the energy dissipated by viscosity, *μ*, and is given as a function of hydraulic resistance and flow rate. The subscripts 1 and 2 represent the inlet and the outlet, respectively. This specific form of the Bernoulli's equation is valid, if both Re number and Mach number are estimated to be sufficiently lower than the values where flow becomes turbulent and compressible, respectively. Moreover, flow in the device is non‐uniform because the channel deformation becomes smaller as gas flows from the inlet to the outlet, as shown in Figure S8, Supporting Information. Since Δ*p* = *p*_1_ − *p*_2_, and the effect of gravity is negligible, Equation (4) becomes

(5) $$\Delta p=\frac{1}{2}\rho \left(v_{2}^{2}-v_{1}^{2}\right)+w_{\mu }$$

with

(6) $$w_{\mu }=\frac{105\mu Ql}{4wh^{3}}$$

where *Q* is the flow rate, *l* is the length of the channel, *w* is the width of the channel, and *h* is the height of the channel. These specific numerical constants in this expression for *w*_*μ*_ arise from the parabolic cross section used here to estimate Δ*p* in the channel.^[^ ^22^ ^]^ We denote the PDMS displacement in *z*‐direction at the inlet and the outlet as *h*_1_ and *h*_2_ and tentatively use 500 and 100 µm, respectively, based on the results shown in Figure 4c, as well as, Figure S8, Supporting Information, that shows *h*_2_ is several times smaller than *h*_1_. To calculate *v*_1_ and *v*_2_, we make the simplifying assumption that the cross‐sectional shape is a triangle and assume that

(7) $$h=\left(h_{1}+h_{2}\right)/2$$

Remarkably, we nearly exactly reproduce the experimental data, even including the seemingly randomly scattered variations, as shown in Figure 4d. By contrast, if we calculate the values using Bernoulli's equation without *w*_*μ*_, we obtain a linear relationship between Δ*p* and density that deviates significantly from the experimental results, as shown in Figure S9, Supporting Information. By examining the results, we see that the random behavior of the data reflects the summation of the kinetic energy and the viscous loss of each gas that is dependent on two gas‐specific parameters, density and viscosity, which vary independently of one another, as listed in Table 1. We also find that Δ*p* depends linearly on the displacement, as shown in Figure 4e. Thus, the correlation between gas properties, PDMS displacement, and color change enables us to determine the physical properties of gases based on the color patterns of the proposed device.

**Figure 5:**
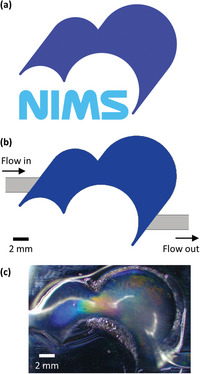
Flow‐induced display of a logo. a) The NIMS logo. b) Schematic of the Ar plasma‐treated area whose shape is exactly the same as that shown in (a). A gas flows from the left to the right. c) Photo of the device under the flow of N_2_ at 10 mL min^−1^.

As the change in color occurs when any gas flows through the Ar‐plasma treated area, this technique can be used to display an arbitrary pattern below the flow. To demonstrate this concept, we fabricate a device using a mask whose shape is the same as that of an institute logo, as shown in **Figure** **5**a,b. When N_2_ is flowed at 10 mL min^−1^, it deforms the PDMS almost exactly in the shape of the logo, exhibiting a brilliant color, as shown in Figure 5c.

The dependence of the color intensity on the different parameters of the gases is determined by the change in pressure required to achieve the desired flow rate, and the amount of deformation, and hence color change, obtained for the pressure required to achieve the set flow rate. While this dependence is non‐linear and quite complex, the pattern does have sufficient details that make it possible to discriminate the gases and their properties using advanced analytical techniques such as machine learning. Detailed analysis of gases and their discrimination will be also possible, even with a compact setup, by coupling the present device with another one that enables us to determine density or viscosity.^[^ ^23^ ^]^

## 3 Conclusion

We measure flow of gases using a device that consists of a glass slide and a PDMS slab whose surface is locally treated with Ar and O_2_ plasma. We use Ar plasma to obtain the PDMS surface whose optical transmittance in the visible range changes in response to strain. This optical change in the form of structural color is due to the formation of well‐defined wrinkles on the surface of the PDMS under the strain. Unlike an O_2_ plasma‐treated PDMS, the Ar plasma‐treated PDMS does not bond to a glass surface. Thus, by using a mask, we obtain a PDMS slab where only part of the surface is treated with an Ar plasma while the rest is treated with an O_2_ plasma. In this way, we fabricate a PDMS device that has no pre‐formed channel but is capable of flowing gas only between the Ar plasma‐treated area and the glass surface. Since a gas flow deforms the PDMS, it induces a change in color that is correlated with flow rate, viscosity, and density of the gases used, including He, Ne, N_2_, Ar, CO_2_, and Xe. Here, we mainly aim to demonstrate the feasibility of measuring gases using this very simple device. Future investigation will explore the dependence on many factors including the thickness of PDMS and its Young's modulus as these can be tuned to more accurately measure the parameters of the flow over a broader range. We also demonstrate that this technique is applicable to the flow‐induced display of an arbitrary pattern such as a logo. Since the pattern shows a complicated color gradation that reflects the consequences of several parameters of gases, machine learning‐based analysis will enable us to discriminate various gaseous mixtures such as odors. The present approach to the gas measurement and analysis will contribute not only to a wide range of scientific applications, including fluid sensing and display, but also to fields of art and entertainment.

## 4 Experimental Section

### Preparation of Polydimethylsiloxane

A kit including PDMS base and curing agent (Sylgard 184, Dow Corning) was used to prepare PDMS slabs. The liquid PDMS mixture, consisting of the base and the curing agent with a weight ratio of 30:1, was degassed and poured into a petri dish. After curing overnight at 65 °C, the PDMS was cut into pieces for further experiments.

### Plasma Treatments

A low‐pressure plasma system (Femto, version B) was purchased from Diener Electronic GmbH + Co. KG. Argon was used as a plasma source to obtain the PDMS with strain‐induced wrinkles. Oxygen was used as the other plasma source for creating an active surface for bonding with a glass substrate. The area to which the plasma was applied was controlled by using a 3D‐printed mask. A piece of PDMS with the mask was loaded in the plasma chamber and treated with the plasma power, pressure, and duration being 100 W, 0.6 mbar, and 1 min, respectively.

### Device Fabrication

A PDMS slab (20 mm × 50 mm × 2.5 mm) was used to fabricate a channel‐free device. The procedure was schematically shown in Figure 1a. To make an inlet and an outlet, two holes (1.5 mm in diameter) were punched at both ends of an area that was exposed to an Ar plasma. A mask with a rectangular opening (25 mm × 10 mm) was used to cover an area which was exposed to an O_2_ plasma later. The PDMS slab was stretched with 20% strain for a few seconds soon after the Ar plasma treatment. When the PDMS slab was exposed to an O_2_ plasma, a negative mask was used to cover the Ar plasma‐treated area. A glass substrate was also loaded in the plasma chamber to activate its surface by an O_2_ plasma. The conditions for this plasma treatment were 35 W, 0.6 mbar, and 20 s. The two plasma‐treated PDMS was then adhered to the glass substrate. The device was put into an oven and was heated at 65 °C for a few minutes to enhance the interfacial bonding.

### Surface Imaging under Compression Using Molds

The Ar plasma‐treated PDMS (20 mm × 50 mm × 2.5 mm) was adhered to the surface of a 3D‐printed mold as shown in Figure 3b. To control strain, three molds with different curvatures including 5, 10, and 20 m^−1^ were fabricated_._ These numbers corresponded to strains of 0.8%, 1.3%, and 2.5%, respectively. The samples were observed by using a 3D surface profiler (VK‐X3000, KEYENCE Corporation) under the laser confocal mode.

### Gas Measurements

He, Ne, N_2_, Ar, CO_2_, and Xe were used, and the flow was regulated with a mass flow controller (MFC; SEC‐N112MGM, Horiba Ltd.). The program for controlling MFC was designed using LabVIEW (NI Corporation). The flow was injected through the inlet at various flow rates including 5, 10, 20, 50, 100, 200, 300, and 400 mL min^−1^. The flow rates were measured with a volumetric flow meter (ProFLOW 6000 Electronic Flowmeter, Restek Corporation) at the outlet to confirm that there was no leakage. The device was observed with a stereo microscope (Leica S9i, Leica Microsystems) under flow for the color analyses. The flow‐induced change in color of the device was analyzed using ImageJ software (version 1.53k).^[^ ^24^ ^]^

### Finite Element Analysis Simulation

The deformation of PDMS under various gas flow conditions was modeled in the FEA software COMSOL Multiphysics 5.6 as a time‐dependent problem. The laminar flow and the structural mechanics interfaces were used and set to be fully coupled, such that the fluid flow‐induced PDMS deformation will provide feedback to the laminar flow interface via a mesh update, which alters the flow profile and changes the PDMS deformation iteratively until convergence. Because of the difficulty in modeling a tightly adhered interface being peeled open by an internal gas flow, the bulging phenomenon was modeled as the deformation of a narrow channel with its height of 50 µm under an N_2_ flow at 400 mL min^−1^, assuming there was no contact force at the interface. The PDMS channel was 10 mm in width and 25 mm in length, with a top wall thickness of 2.5 mm. More details on the numerical model were described in a previous paper^[^ ^16^ ^]^ where the deformation of a smaller channel was calculated and verified by experiments.

## Conflict of Interest

The authors declare no conflict of interest.

## Supporting information

Supporting Information

## Data Availability

The data that support the findings of this study are available from the corresponding author upon reasonable request.

## References

[1] a) L. Fusnik , B. Szafraniak , A. Paleczek , D. Grochala , A. Rydosz , Sensors 2022, 22, 2557; 35408172 10.3390/s22072557 PMC9002727

[2] a) C. Chi , X. Sun , N. Xue , T. Li , C. Liu , Sensors 2018, 18, 948; 29565835 10.3390/s18040948 PMC5948515

[3] a) T. Mizukaki , F. Iwasaki , M. Mori , A. Kato , D. Numata , Sci. Technol. Energ. Mater. 2021, 82, 95;

[4] a) K. Hosokawa , K. Hanada , R. Maeda , J. Micromech. Microeng. 2002, 12, 1;

[5] A. Orth , E. Schonbrun , K. B. Crozier , Lab Chip 2011, 11, 3810. 21964718 10.1039/c1lc20114j

[6] W. Z. Song , D. Psaltis , Biomicrofluidics 2011, 5, 044110. 10.1063/1.3664693 PMC3364809 22662062

[7] C. H. D. Tsai , M. Kaneko , Biomicrofluidics 2016, 10, 024116. 27076864 10.1063/1.4945412 PMC4818274

[8] C. Hoera , A. Kiontke , M. Pahl , D. Belder , Sens. Actuators, B 2018, 255, 2407.

[9] J. M. K. Ng , I. Gitlin , A. D. Stroock , G. M. Whitesides , Electrophoresis 2002, 23, 3461. 12412113 10.1002/1522-2683(200210)23:20<3461::AID-ELPS3461>3.0.CO;2-8

[10] a) K. L. Mills , X. Zhu , S. Takayama , M. D. Thouless , J. Mater. Res. 2008, 23, 37; 19779588 10.1557/JMR.2008.0029 PMC2749279

[11] M. J. Owen , P. J. Smith , J. Adhes. Sci. Technol. 1994, 8, 1063.

[12] a) J. Bacharouche , P. Kunemann , P. Fioux , M.‐F. Vallat , J. Lalevée , J. Hemmerlé , V. Roucoules , Appl. Surf. Sci. 2013, 270, 64;

[13] A. L. Volynskii , S. Bazhenov , O. V. Lebedeva , N. F. Bakeev , J. Mater. Sci. 2000, 35, 547.

[14] P. Görrn , S. Wagner , J. Appl. Phys. 2010, 108, 093522.

[15] N. Bowden , S. Brittain , A. G. Evans , J. W. Hutchinson , G. M. Whitesides , Nature 1998, 393, 146.

[16] T. Gervais , J. El‐Ali , A. Günther , K. F. Jensen , Lab Chip 2006, 6, 500. 16572212 10.1039/b513524a

[17] F. Brau , H. Vandeparre , A. Sabbah , C. Poulard , A. Boudaoud , P. Damman , Nat. Phys. 2011, 7, 56.

[18] S. Befahy , P. Lipnik , T. Pardoen , C. Nascimento , B. Patris , P. Bertrand , S. Yunus , Langmuir 2010, 26, 3372. 19947617 10.1021/la903154y

[19] I. D. Johnston , D. K. McCluskey , C. K. L. Tan , M. C. Tracey , J. Micromech. Microeng. 2014, 24, 035017.

[20] Handbook of Chemistry and Physics, CRC Press, 2014, pp 6–229.

[21] C. E. Synolakis , H. S. Badeer , Am. J. Phys. 1989, 57, 1013.

[22] H. Bruus , Lab Chip 2011, 11, 3742. 22011885 10.1039/c1lc20658c

[23] a) K. Shiba , G. Li , E. Virot , G. Yoshikawa , D. A. Weitz , Lab Chip 2021, 21, 2805; 34105583 10.1039/d1lc00202c

[24] W. S. Rasband , ImageJ, http://imagej.nih.gov/ij/, 1997.

