# Supplementary material for: Visualization of Flow‐Induced Strain Using Structural Color in Channel‐Free Polydimethylsiloxane Devices
Source: Adv Sci (Weinh). 2022 Nov 17;10(1):2204310. doi: 10.1002/advs.202204310 (PMC9811489; doi:10.1002/advs.202204310)
Supplement: Supplementary file 1 — Supporting Information [file ADVS-10-2204310-s001.pdf]

Supporting Information

Visualization of flow-induced strain using structural color in channel-free PDMS devices

Kota Shiba, Chao Zhuang, Kosuke Minami, Gaku Imamura, Ryo Tamura, Sadaki Samitsu, Takumi Idei, Genki Yoshikawa, Luyi Sun, and David A. Weitz**

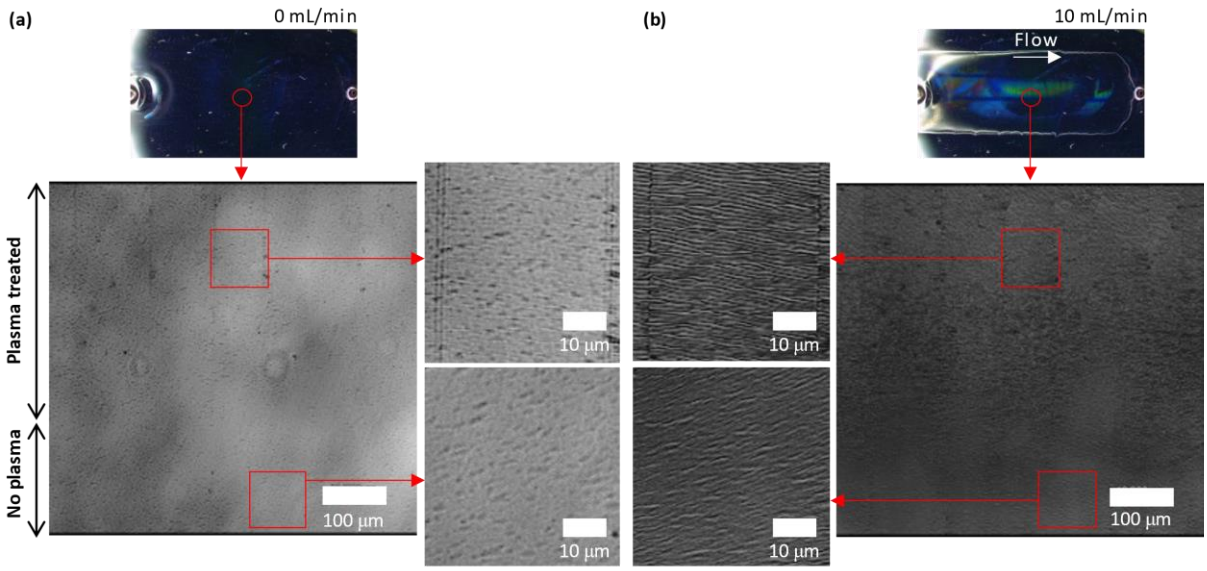

Figure S1. In situ observation of the inner top wall of the device (a) without and (b) with N_2 flow at 10 mL/min. The boundary area that includes both plasma treated and no plasma areas is captured. Close-up images of each area marked with the red squares are also shown.

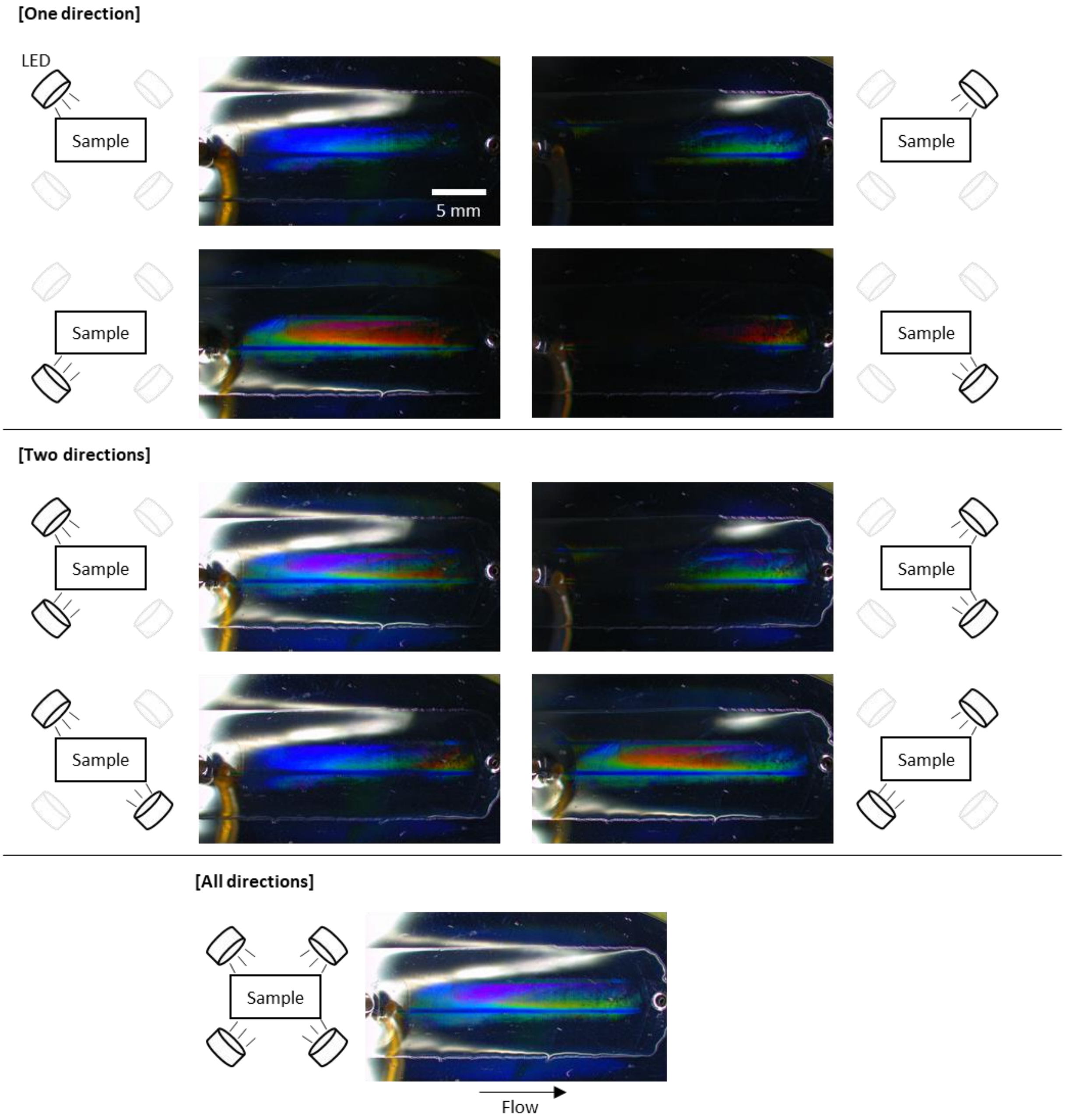

Figure S2. Photos of the device taken under different lighting conditions. N_2 is flowed at 400 mL/min.

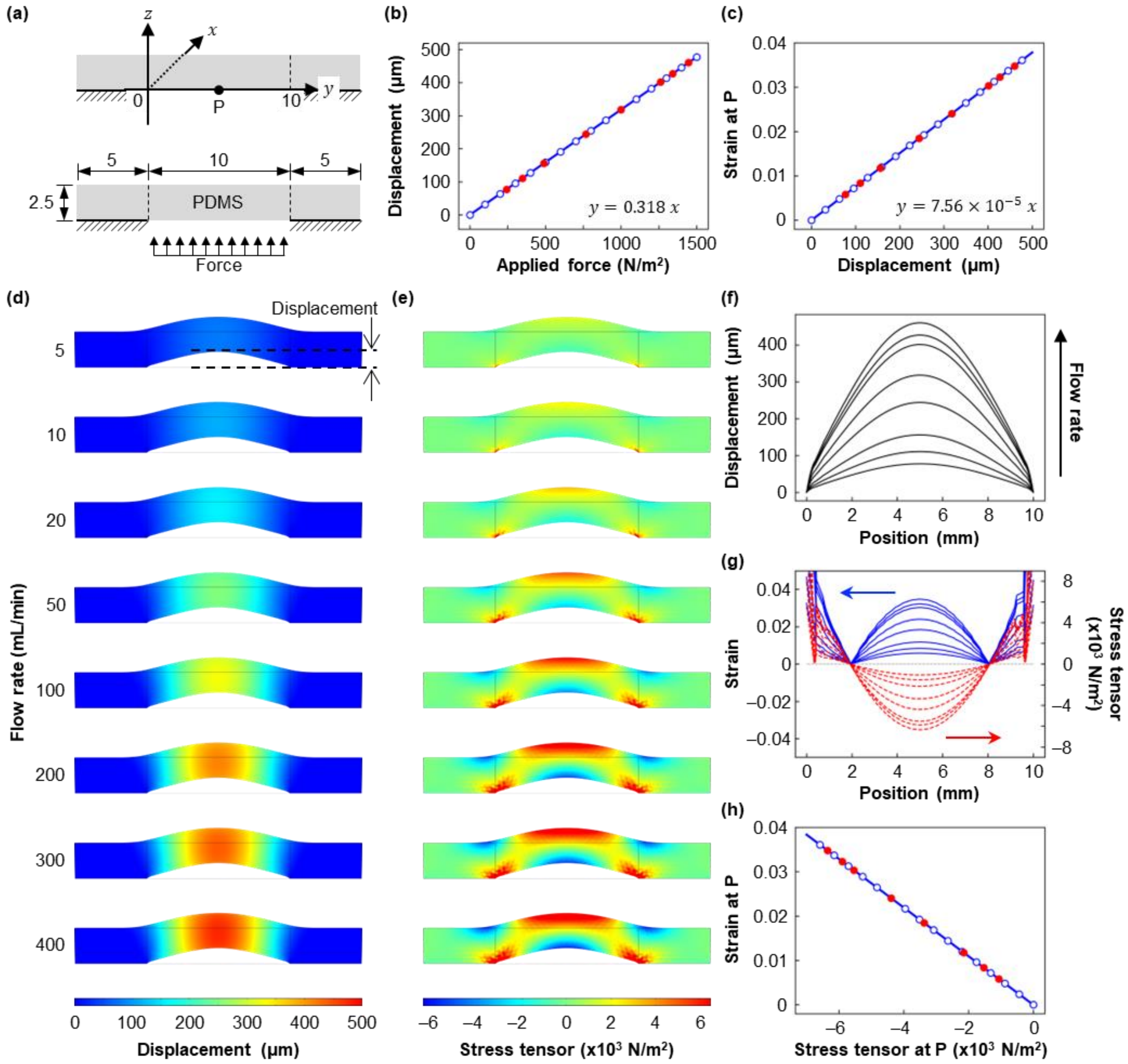

Figure S3. FEA simulation of strain-displacement relationship. (a) Cross-sectional model of a PDMS slab. Cartesian coordinates of the model and configuration and fixed constraint of the model are shown on the top and the bottom, respectively. Uniformly distributed force is applied on the bottom free surface of the PDMS. (b) Plot of simulated displacement as a function of applied force. The eight red points are plotted based on the experimentally obtained displacements that are shown in Figure 2(e). (c) Plot of simulated strain as a function of simulated displacement. The eight red points are also plotted based on the experimentally obtained displacements that are shown in Figure 2(e). (d, e) Mapping of simulated displacement and stress tensor over the PDMS. Each result shows the simulated displacement and the stress tensor that are expected under the flow of N_2 at 5, 10, 20, 50, 100, 200, 300, and 400 mL/min. (f) Simulated profile of the bottom surface of the PDMS. (g) Profile of simulated strain (blue) and stress tensor (red) along the bottom surface of the PDMS. (h) Plot

of simulated strain as a function of stress tensor. The eight red points are plotted based on the simulated strain values estimated in (c) that correspond to the experimentally obtained displacements shown in Figure 2(e).

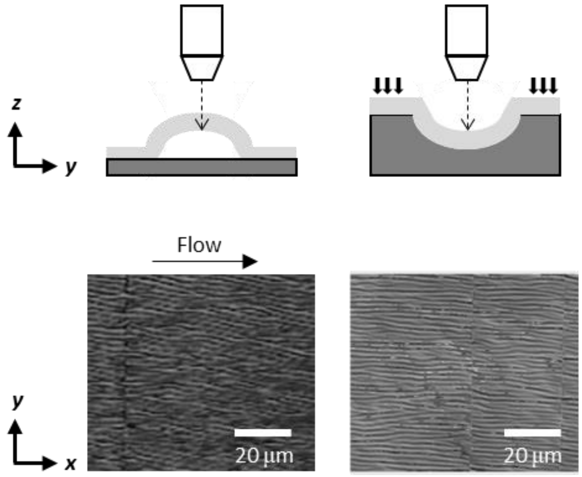

Figure S4. Comparison of the wrinkles formed by two different approaches: (left) under the flow of N_2 at 10 mL/min and (right) under the compression using the curved mold.

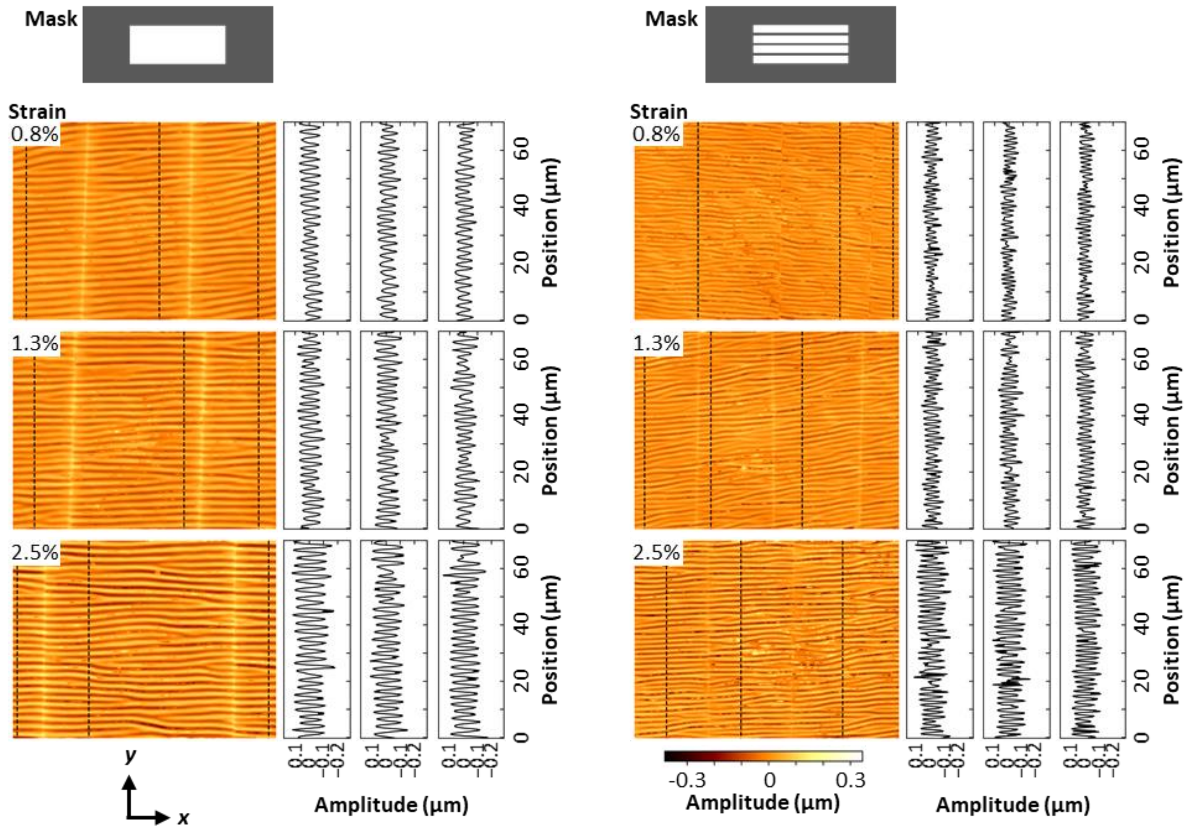

Figure S5. Images of the PDMS slabs taken under the strain of 0.8%, 1.3%, and 2.5% using molds with different curvatures. Three amplitude profiles are recorded along the dashed lines in each image. The left three and right three data are obtained from two PDMS slabs that are plasma-treated with different masks: (left) without and (right) with lines.

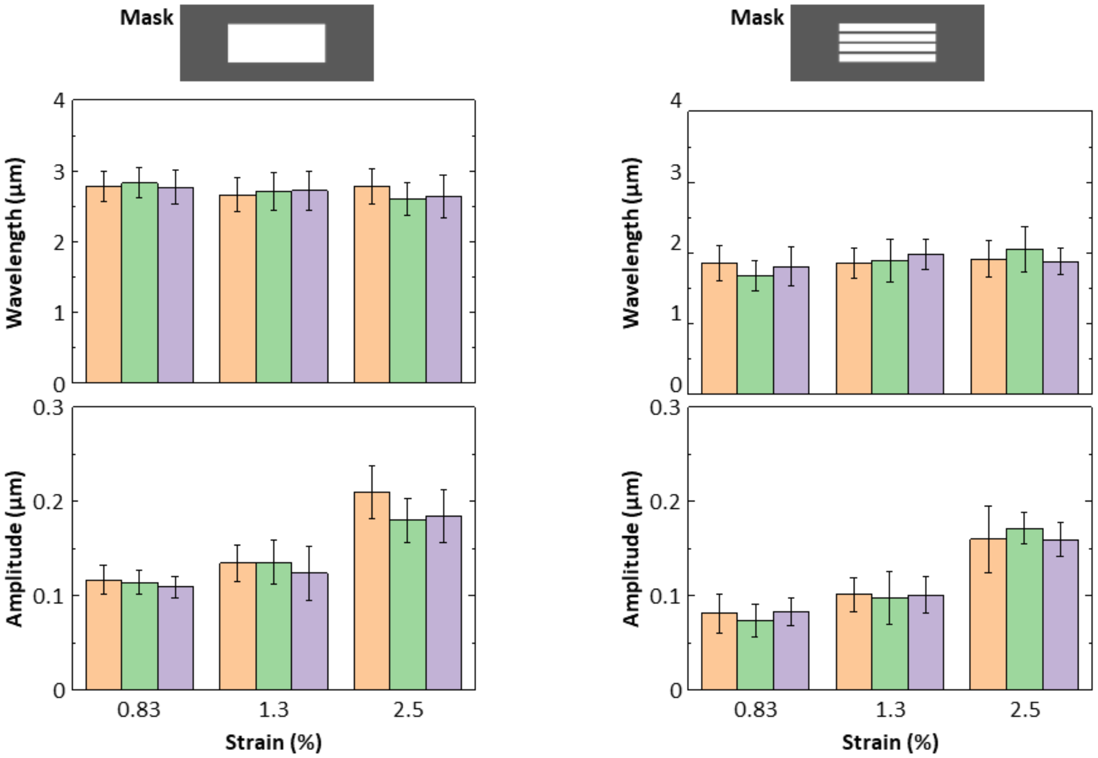

Figure S6. The relationship between wavelength (top) and amplitude (bottom) as a function of strain. The left two and right two data are obtained from two PDMS slabs that are plasma-treated with different masks: (left) without and (right) with lines. The three amplitude profiles in each image shown in Figure S5 are used to draw the bar graphs. The three colors (orange, green, and purple) correspond to the amplitude profiles from the left to the right in Figure S5.

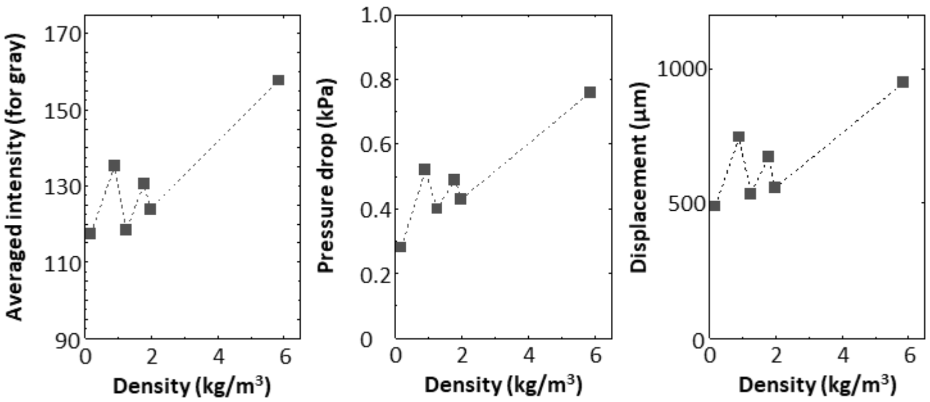

Figure S7. Plot of averaged intensities for gray (left), pressure drops (middle), and displacements (right) as a function of densities

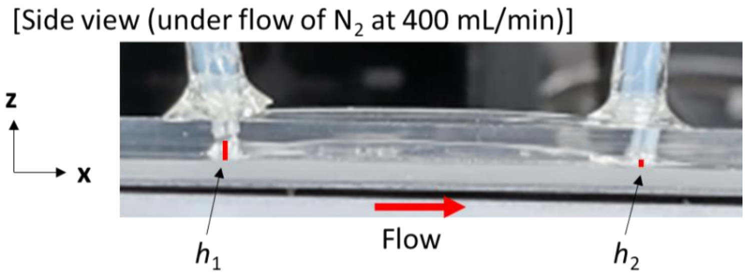

Figure S8. Photo (side view) of the PDMS device taken under the flow of N_2 at 400 mL/min.

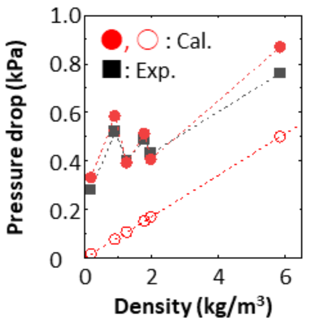

Figure S9. Plot of pressure drops as a function of densities. The black squares and the red circles (open and closed) show experimental data and calculated values, respectively. The values for the closed and open circles are calculated using Bernoulli's equation with and without the viscous loss term, respectively.
